# Single cell atlas of kidney cancer endothelial cells reveals distinct expression profiles and phenotypes

**DOI:** 10.21203/rs.3.rs-3558517/v1

**Published:** 2023-11-10

**Authors:** Yuexin Xu, Chris P. Miller, Jun Xue, Ying Zheng, Edus H. Warren, Scott S. Tykodi, Shreeram Akilesh

**Affiliations:** Fred Hutchinson Cancer Center; Fred Hutchinson Cancer Center; University of Washington; University of Washington; Fred Hutchinson Cancer Center; University of Washington; University of Washington

**Keywords:** Renal cell carcinoma, Endothelial cells, Single-cell RNA sequencing, Immune cell adhesion

## Abstract

**Background:**

Tumor endothelial cells (TECs) represent the primary interface between the tumor microenvironment and circulating immune cells, however their phenotypes are incompletely understood in highly vascularized clear cell renal cell carcinoma (ccRCC).

**Methods:**

We purified tumor and matched normal endothelial cells (NECs) from ccRCC specimens and performed single-cell RNA-sequencing to create a reference-quality atlas available as a searchable web resource for gene expression patterns. We established paired primary TECs and NECs cultures for ex vivo functional testing.

**Results:**

TECs from multiple donors shared a common phenotype with increased expression of pathways related to extracellular matrix regulation, cell-cell communication, and insulin-like growth factor signaling that was conserved in comparison to hepatocellular carcinoma associated TECs, suggesting convergent TEC phenotypes between unrelated tumors. Cultured TECs stably maintained a core program of differentially regulated genes, were inherently resistant to apoptosis after vascular endothelial growth factor removal and displayed increased adhesiveness to subsets of immune cells including regulatory T-cells.

**Conclusions:**

Our studies delineate unique functional and phenotypic properties of TECs, which may provide insights into their interactions with available and emerging therapies. Functional phenotypes of cultured TECs suggest potential mechanisms of resistance to both antiangiogenic and immune-based therapies.

## Introduction

Clear cell renal cell carcinoma (ccRCC) is the most common malignant tumor of the kidney in adults. The majority of spontaneously arising ccRCC tumors exhibit inactivation of the von Hippel-Lindau tumor suppressor gene, which in turn stabilizes the hypoxia-inducible factor (HIF) signaling pathway (1–5). Constitutive HIF activity stimulates a pro-angiogenic program, including overexpression of vascular endothelial growth factor (VEGF) (6) and as a result, ccRCC tumors are highly vascularized. Tyrosine kinase inhibitor (TKI) mediated blockade of angiogenic signaling via targeting of VEGF-receptors remains a cornerstone of medical therapy for patients with these tumors (7, 8).

ccRCC tumors are often immune-infiltrated (9) and responsive to immune checkpoint inhibitors, which are now administered in combination with TKIs as primary systemic therapy for advanced disease (10, 11). Tumor endothelial cells (TECs) and cell-cell junctions are recognized as the gateway for host immune cells entering the tumor microenvironment. Previous studies of TECs using single-cell RNA-seq (scRNA-seq) in various cancers have revealed significant differences in TEC gene expression and functional phenotypes as compared to their normal endothelial cell (NEC) counterparts (12–15). Despite the highly vascular nature of ccRCC, previous analyses of TECs in ccRCC are limited to scRNA-seq performed on the entire tumor mass in which TECs represented only a small fraction of the total input cell number (12–14). Without specific enrichment for TECs, this limited sampling represents an underpowered analysis of the TEC phenotype (16, 17). In addition, not all ccRCC studies included comparative analysis to matched NECs. Furthermore, none of the prior analyses of TEC from ccRCC assessed the stability of TEC phenotypes in *ex vivo* primary culture, their barrier function regulating entry of immune cells into the tumor, and their putative role in shaping the infiltrating immune cell phenotype of the tumor microenvironment (18–20).

Therefore, in this study, we purified TECs and NECs from ccRCC nephrectomy tissues to perform an in-depth characterization of their gene expression patterns and phenotypes. We validated that TECs from ccRCC have a phenotype that is distinct from NECs. We found a congruent expression signature for TECs from ccRCC when compared with those from hepatocellular carcinoma, a disease similarly responsive to anti-angiogenic and immune checkpoint inhibitor therapies. We also revealed that many expression programs of TECs were stable in *ex vivo* culture. Cultured TECs displayed tolerance to VEGF withdrawal and enhanced binding to autologous leukocytes, especially T-cells and monocytes. Our analysis platform can be exploited for further study of therapeutic targeting of TECs across tumor types as well as for insight into immune cell binding interactions with TECs, the first component of the tumor microenvironment encountered by immune cells.

## Methods

### Study cohort

We collected four paired tumor, normal kidney and PBMC biospecimens from treatment naive patients who underwent partial or full nephrectomy surgery (Table S1). The tumor histology was confirmed as ccRCC for all four patients on their final pathology report.

### Single-cell RNA-sequencing

Tissue single-cell suspensions were stained by DAPI, 1:20 dilution of PE-labeled Mouse anti-Human CD144, and APC anti-human CD31 (clone WM59, Biolegend, San Diego, CA) and sorted by flow cytometry (FACSAria III, BD biosciences). CD144^+^DAPI^−^ ECs and DAPI-live cells from each sample were sorted into two separate tubes and labeled by TotalSeq^™^-C0251 anti-human Hashtag 1 Antibody (HTO-1, Biolegend) and HTO-2 respectively according to manufacturer’s protocol. The sorted cells were then combined at 2:1 ratio. 5,000 DAPI-HUVEC cells labeled with HTO-3 were spiked into each of the samples. Samples were then loaded at 17,000 cells per lane onto a 10X Genomics Controller (10X Genomics, Pleasanton, CA). scRNA-seq libraries were constructed according to the manufacturer’s protocol. Pooled V(D)J, 5′ GEX libraries and HTO libraries were sequenced on a NovaSeq 6000 SP100 flowcell (Illumina, San Diego, CA) to obtain 5,000 reads/cell, 20,000 reads/cell, and 5,000 reads/cell depth, respectively.

### Primary EC culture

EC media were made with EBM-2 base media (Lonza, Basel, Switzerland) with 1% anti-anti (Thermo Fisher Scientific, Waltham, MA) and 10% FBS (ThermoFisher Scientific, Waltham, MA), supplement with 500x ECGS (Cell Biologics, Chicago, IL) and 50ug/ml Heparin (Thermo Fisher Scientific). To enrich ECs, human EpCAM microbeads (Miltenyi Biotec) were used to deplete epithelial cells from single-cell suspensions. The EC cultures were maintained in recombinant human VEGF-165 (PeproTech, Rocky Hill, NJ) at 40 ng/ml and 1% low O2 condition 8 days. The passage one cultures were purified further by 1:20 dilution of PE-labeled Mouse anti-Human CD144 (clone 55-7H1; BD Biosciences, San Jose, CA) sorting and then culture for two passages with 20 ng/μl human VEGF containing EC media at the same low O2 condition.

For VEGF retrieval experiments, NAT and tumor derived ECs were stained by CellTracker^™^ Red CMTPX Dye (Thermo Fisher Scientific) at 37 degrees for 30 minutes, then maintained in the presence or absence of 20ng/mL VEGF containing media. Two days after the culture, the floating cells were harvested, and the plate were imaged. The rest of adherent cells were harvested and combined with the floating cells. Overall viability was assessed by DAPI staining by flow cytometry. The % of confluency was measured by Fiji ImageJ (21) on the image stacks by applying macro functions first set cell boarder threshold to parameter and then measure the %Area of each frame.

### RNA in situ hybridization (RNA-ISH)

5μm FFPE sections of RCC tumor and matched NAT from the 4 donors used for scRNA-seq were used for RNA-ISH using ACDBio’s primary probes targeting IGFBP3 (Catalog # 310351, ACDBio, Newark, CA) and IGFBP5 (Catalog # 452381, ACDBio) and the RNAScope 2.5 HD-RED development kit (Catalog # 322350, ACDBio). After colorimetric development, slides were cover-slipped with EcoMount (BioCare Medical, Pacheco, CA) and imaged on an Olympus BX41 upright microscope (Tokyo, Japan) equipped with a Leica DFC420 camera (Leica, Wetzlar, Germany).

### Bioinformatics and statistics

The 10X Chromium scRNA-seq outputs were de-multiplexed, mapped to the human reference genome (hg19, GRCh38) and aggregated to one single-cell object through the Cell Ranger V4.0 bioinformatics pipeline (10X Genomics). Batch effect corrections were performed as described (22). Dimension reduction and clustering of 10X scRNA-seq data were conducted using the Seurat package (23). The pseudotime analyses were conducted through the Monocle3 package (24–26). The cell classification was performed by Garnett R package (27) based on marker genes markers (Table S2). DEGs were identified using the nonparametric Wilcoxon rank sum test by FindMarkers function of Seurat package to find DEGs from each identity cluster against the remaining cells, with min.pct = 0.25, logfc.threshold = 0.25. We used default options for the analysis if not specified otherwise. DEG results were visualized using EnhancedVolcanoR package (version 1.10.0) https://github.com/kevinblighe/EnhancedVolcano. FeaturePlot, DimPlot, and DotPlot functions of the Seurat package were used for visualization of selected genes. The ‘VlnPlot’ function of Seurat package was used for violin plots to show the expression level of selected genes with log normalized value by default. Pathway analysis were conducted using R package clusterProfiler (28).

Raw RNAseq data were processed by the nextflow nf-core/rnaseq analysis pipeline using STAR, RSEM, HISAT2 or Salmon with gene/isoform counts and extensive quality control (29). DEGs were calculated by DEseq2 R package (30). Pathway analysis were conducted using R package clusterProfiler (28). Proportional Venn diagram of preserved DEGs was plotted using BioVenn R package (31).

### Other methods

Other methods are included in the Supplementary Methods.

## Results

### TECs from ccRCC tumors have a gene expression profile distinct from NECs isolated from renal cortex.

To characterize the expression phenotype of TECs from ccRCC and to compare to their normal counterpart (NEC) isolated from normal adjacent renal cortex, we performed scRNA-seq on TECs and NECs purified by flow cytometry from four different donor nephrectomies. The four TEC/NEC sample pairs were batched into two separate scRNA-seq libraries. We spiked in human umbilical vein endothelial cells (HUVECs) in all samples allowing us to study batch-to-batch variation between libraries. TECs/NECs, HUVECs and an aliquot of the entire tumor/normal adjacent tissue (NAT) were surface-labeled with unique hashtag oligonucleotide (HTO)-labeled antibodies targeting both human CD298 and β2 microglobulin prior to pooling. The oligonucleotide barcodes attached to the HTO-labeled antibodies remained associated with the cells during library generation. We utilized the readout of these HTO-antibodies as an independent measure of cellular identity at the analysis stage (see below).

**Figure 1:**
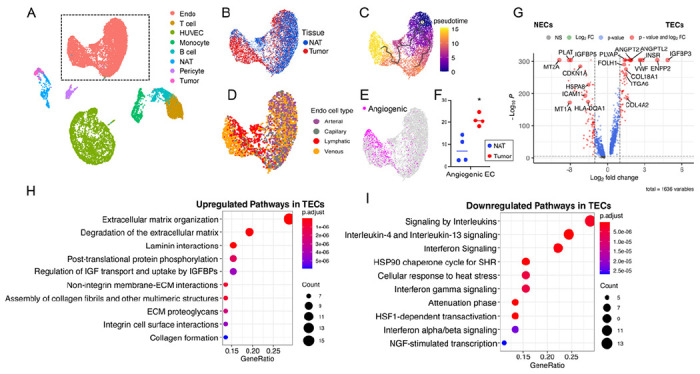
Analysis of scRNA-seq transcriptome data for TECs and NECs from ccRCC patients. **(A)** UMAP plot of scRNA-seq dataset from 4 subjects colored by clusters. The dashed line marks the endothelial cell cluster. UMAP plots marked by **(B)** tissue origin, **(C)** pseudotime trajectory, **(D)** general EC lineages and **(E)** angiogenic ECs. **(F)** Quantification of angiogenic NECs and TECs numbers. Each dot represents one patient. Statistics: *, P < 0.05, t-test. **(G)** Volcano plot of DEGs in TECs compared with NECs. Dashed lines, cutoffs: P < 1x10^−5^; FC = 1. **(H)** Upregulated reactome pathways in TECs with DEGs at log2FC ≥ 1 compared with NECs. **(I)** Upregulated reactome pathways in NECs with DEGs at log2FC ≥ 1 compared with TECs.

Uniform Manifold Approximation and Projection (UMAP) projections showed distinct cell types clustered according to gene expression patterns (Fig. 1A). Using the HTO-barcodes as an orthogonal measure of cell identity, we saw clear separation of isolated ECs (HTO-antibody1) from non-EC cell populations (HTO-antibody2) within tumor/NAT digests and from HUVECs (HTO-antibody3) (Suppl. Figure 1A-C). In addition, The HTO-antibody3 labeled HUVEC populations were overlapped between different libraries indicating minimum batch effect, suggesting that there was no observed bias in representation of cells as a function of donor or sequencing library (Suppl. Figure 1B, D, E). Furthermore, expression of the *ACTB* housekeeping gene was uniform across the different clusters, consistent with comparable transcript counts/sample over the different cell types (Suppl. Figure 1F). Overall, after quality control and filtering, our dataset consisted of 14,982 total cells (library1: 3,630 cells and library2: 11,352 cells), of which, 7,512 cells were TECs or NECs (library1: 2,467 ECs and library2: 5,045 ECs).

Further exploration of the UMAP projection revealed distinct cell type clusters (ECs, immune cells, tumor cells, normal kidney epithelial cells, and the control HUVEC clusters, Fig. 1A) that expressed expected cell-defining markers (Suppl. Figure 1G) confirming the cell type assignment. For example, ECs expressed *PECAM1* (CD31) and *CDH5* (VE-Cadherin). Though HUVECs expressed EC markers, their overall expression pattern was distinct from primary NECs and TECs. Focused analysis of the primary EC cluster showed a clear separation of TECs and NECs as well as a transitional population with an intermediate phenotype (Fig. 1B). Pseudotime trajectory analysis indicated a possible developmental trajectory from NECs towards TECs (Fig. 1C). We then classified the ECs according to known phenotypes described as a function of their anatomic location (32) (Table S2). This analysis revealed that NECs from the kidney have a heterogeneous mixture of venous, arterial, capillary, and lymphatic vasculature phenotypes (33–35), whereas TECs predominantly exhibited a venous EC phenotype (Fig. 1D). Cell type classification using a set of angiogenic EC markers (GO angiogenic pathway 0001525 (36, 37)) also revealed that TECs were more likely to exhibit an angiogenic phenotype than NECs (Fig. 1E and F). Taken together, these analyses confirmed that TECs have a phenotype that is distinct from NECs.

To explore the basis of this distinct TEC phenotype, we performed differentially expressed gene (DEG) analysis. A total of 1,636 genes were differentially expressed when comparing TECs with NECs (Table S3). The top 3 DEGs highly expressed in TECs versus NECs were *IGFBP3, ENPP2 and INSR*, whereas *MT2A, PLAT* and *IFGBP5* were the top 3 genes preferentially expressed in NECs (Fig. 1G). Pathway analysis of the DEGs identified extracellular matrix (ECM) organization, laminin interactions, collagen fibrils assembly, non-integrin membrane-ECM interactions, integrin cell surface interactions and IGF regulation as functional pathways upregulated in TECs (Fig. 1H). Exploration of individual matrix metalloproteinase (MMPs) and a disintegrin and metalloproteinase with thrombospondin motifs (ADAMTS) family members also identified several genes with increased expression in TECs (Suppl. Figure 2A & B). These included enzymes with known roles in promoting angiogenesis such as *MMP9* (38), *MMP14* (39) and *ADAMTS13* (40). Conversely, in comparison to NECs, TECs had reduced expression of pathways involved in immune regulation such as signaling by interleukins and interferon gamma signaling (Fig. 1I).

### Identification of IGFBP3 as a marker gene for TECs

**Figure 2:**
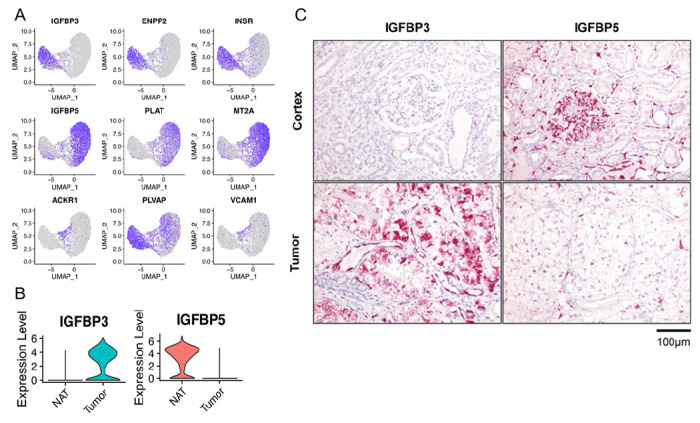
*IGFBP3* and *IGFBP5* expression in TECs and NECs. **(A)** The top three DEGs in TEC, NEC and transitional EC clusters. **(B)** Single-cell expression of *IGFBP3* and *IGFBP5* in TECs and NECs. **(C)** RNA-ISH distribution of *IGFBP3* and *IGFBP5* in tumor and normal kidney tissue. Specific regions of expression are marked.

UMAP projections clearly showed preferential expression of the top 3 DEGs in TECs (*IGFBP3, ENPP2* and *INSR*) and NECs (*IGFBP5, PLAT* and *MT2A*) (Fig. 2A). Previous studies have proposed *IGFBP3, ACKR1* and *PLVAP* as universal TEC marker genes (15). While *IGFBP3* was clearly expressed in TECs, we found that *ACKR1* was instead expressed in a transitional subpopulation of ECs derived mainly from normal kidney tissue with a smaller contribution of ECs isolated from the tumor. *PLVAP* had higher expression in TECs, but NECs also expressed this gene and therefore it did not appear to be a TEC-specific marker. The cell-adhesion molecule VCAM1 has reported expression on a subset of pancreatic TECs (41), though in our dataset, it was localized to the same transitional subpopulation of ECs that expressed *ACKR1*. These UMAP findings confirmed the results of the DEG analysis but also identified differences from previous studies which could be due to the increased resolution of our enriched EC dataset and ccRCC-specific patterns.

Given the consistency of *IGFBP3* as a specific marker of TECs in our dataset and in previous studies (15), we validated its anatomic localization as well as that of its paralog *IGFBP5*, using RNA *in situ* hybridization (RNA-ISH) on formalin-fixed paraffin-embedded (FFPE) sections of RCC and NAT tissues from our 4 donors. *IGFBP3* and *IGFBP5* were two of the highest DEGs between TECs and NECs (Fig. 2B, Table S3). Correlating with the scRNA-seq data, RNA-ISH for *IGFBP3* showed weak expression in rare peritubular capillaries and some tubules, whereas it showed strong expression in the RCC vasculature and patchy expression in the tumor cells themselves (Fig. 2C). By contrast, *IGFBP5* showed strong staining in glomerular and peritubular capillary ECs in normal kidney tissues as well as some arterial smooth muscle cells (*notshown*). *IGFBP5* showed only minimal and patchy expression in RCC tissues. When we examined all the cell populations in our dataset, we confirmed minimal *IGFBP3* expression in RCC tumor cells as well as *IGFBP5* expression in pericytes, which have an overlapping gene expression signature with arterial smooth muscle cells (Suppl. Figure 3A-C). Interestingly, HUVECs were discordant with both TEC and NEC phenotypes demonstrating low levels of both *IGFBP3* and *IGFBP5* expression. Having validated *IGFBP3* and *IGBFP5* as markers of RCC-associated TECs and NECs respectively, we examined the expression profiles of these genes in tumor and normal tissues in The Cancer Genome Atlas (TCGA) dataset. Consistent with our data, *IGFBP3* showed the highest expression in RCC tumors, whereas one of the highest sites of *IGFBP5* expression was normal kidney (Suppl. Figure 3D, E). Increased expression of *IGFBP3* in RCC (and also in lung and colorectal cancer) was associated with inferior overall survival, while *IGFBP5* expression had no apparent impact on prognosis in RCC (Suppl. Figure 3F, G).

### Integration of published RCC scRNA-seq data highlights the advantage of EC enrichment

**Figure 3:**
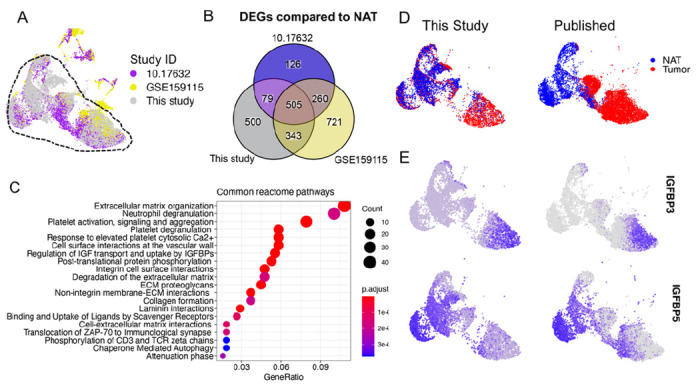
Comparison of EC clusters between previously published ccRCC scRNA-seq and this study. **(A)** UMAP plot of integrated NEC and TEC populations from ccRCC in previously published studies and this study. The dashed line indicates the cells that are clustered in the common clusters. **(B)** Overlap between differentially expressed genes (compared to NAT, cutoffs at logFC = 0.25, minimum detection fraction = 0.1) in two previously published studies and this study. **(C)**Common reactome pathways that are derived from the overlapped DEGs in TECs **(D)**UMAP plot colored by tissue origin of previously published datasets and this dataset **(E)** *IGFBP3* and *IGFBP5* expression in TECs and NECs.

Next, we compared the expression data from this study with previously published scRNA-seq datasets generated from ccRCC. We identified 10 published studies with ccRCC scRNA-seq data, of which only 5 included cells likely to be ECs that were defined by measurable coexpression of *PECAM1* and *CDH5*. None of the prior studies specifically enriched for ECs resulting in an average of 217 ECs/sample (range 2-518/cells sample, Table S4). By contrast, the EC count/sample in this study was 3.7-fold higher than the average published dataset at 939 cells/sample, thus representing the deepest reference dataset for RCC ECs. Of the 5 published studies with EC expression data, 2 did not include paired NAT along with the RCC samples (GSE152938 and GSE171306) and study GSE139555 had only 2 ECs per sample on average. The remaining two published datasets from Li et. al. (10.17632) (42) and Zhang et. al. (GSE159115) (12) included paired TECs and NECs and had adequate EC sampling (207 and 245 cells/sample respectively). We co-clustered these two datasets together with our data to generate a unified UMAP projection (Fig. 3A). 98.4% of all ECs from our samples belonged to cell clusters shared with the published datasets (hashed outline). By contrast, only 66.0% of cells from the 2 published studies could be assigned to the composite cell cluster. The non-overlapping cells showed distinct UMAP localizations even between the two published studies, which may be due to methodologic differences, pre-analytic variation, and/or batch effects. Focusing on the shared cluster of ECs across all 3 datasets, we analyzed DEGs in TECs compared to their matched NECs (Fig. 3B). This identified 505 DEGs that were shared across all 3 datasets. Pathway analyses of this gene list identified significant alterations in ECM organization, neutrophil and platelet degranulation, regulation of IGF transport and uptake by IGFBPs and others (Fig. 3C). Assignment of TECs and NECs to the shared UMAP projection showed representation of almost all EC clusters identified in our samples and in the published studies, though there were some minor differences in represented cell populations (Fig. 3D). Of note, the reciprocal expression of *IGFBP3* and *IGFBP5* in TECs and NECs was reproduced in the combined data (Fig. 3E). This analysis demonstrates that the data from our samples recapitulates major EC clusters seen in previous RCC studies, but also provides a much deeper profiling of matched TEC and NEC expression signatures. The advantage of deeper profiling of isolated ECs is evident since *IGBFP5* was not identified as a marker of NEC in either of the two previous studies and *IGFBP3* was not identified as a TEC marker in one of them (12, 42).

### Congruence of TEC phenotypes between kidney and liver cancer

**Figure 4:**
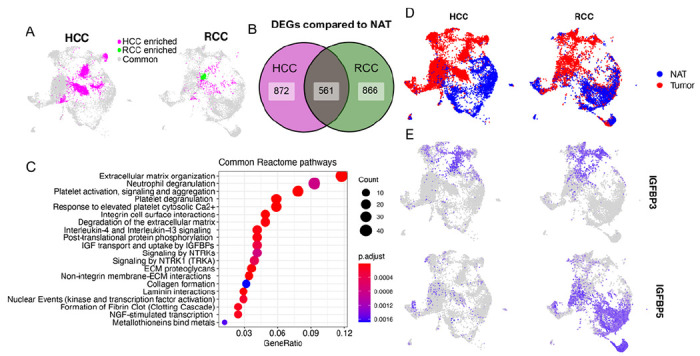
Comparison of EC clusters between the previously published HCC scRNA-seq data and RCC data from this study. **(A)** UMAP plot of integrated TEC populations from HCC and RCC. **(B)** DEG (TECs compared to NECs, cutoffs at logFC = 0.25, minimum detection fraction = 0.1) overlap in the HCC studies and this RCC study. **(C)** Common reactome pathways that are derived from the overlapping DEGs **(D)** UMAP plot colored by tissue origin of ECs from HCC and RCC. **(E)** *IGFBP3*and *IGFBP5* expression in HCC and RCC.

It is not clear if TECs from different types of tumors adopt a unique organ-specific phenotype or a common phenotype that is necessary for tumor growth regardless of tumor location. The second possibility is intriguing since it could lead to therapeutic strategies that target TECs across tumor types. Supporting this concept, a recent study suggested that tip cells from TECs across tumor types adopt a congruent phenotype (43). The deep profiling of ECs enabled by our dataset allowed us to ask a similar question in comparing ccRCC TECs to those from a recently published study on hepatocellular carcinoma (HCC) (20). As we did previously for the RCC scRNA-seq datasets, we combined the scRNA-seq data from hepatocellular carcinoma with our own data to create a unified UMAP projection (Fig. 4A). 92.4% of our RCC TECs overlapped with the HCC TECs and conversely, 75.8% of TECs from the HCC dataset overlapped with TEC clusters also present in this RCC study. While there were TEC populations that were unique to both cancer types, this analysis confirmed that the majority of TECs from both kidney and liver tumors were clustered together and exhibited a congruent expression phenotype (Fig. 4A). By contrast, only 37.3% of NECs from normal kidney overlapped with NEC clusters in liver and reciprocally, 51.7% of NECs from normal liver overlapped with NEC clusters in kidney. This finding suggests that normal tissues have NECs with distinct and organ-specific phenotypes. We then asked which genes were differentially expressed between TECs and NECs in both liver and kidney cancer (Fig. 4B). 561 DEGs were shared between both cancer types and these genes were enriched for many of the same pathways previously seen in RCC TECs alone (Fig. 4C). Mapping onto the combined UMAP projection identified unique NEC and TEC populations in both tissues (Fig. 4D). *IGFBP3* expression remained a marker of both kidney cancer and liver TECs, however *IGFBP5* expression was mainly seen in the kidney and not in liver NECs (Fig. 4E). These analyses showed that while NECs from different organs retained distinct phenotypes that may be important for that organ’s unique functions, by contrast, the majority of TECs from two different tumor types shared a congruent gene expression profile with upregulated pathways related to ECM organization, neutrophil/platelet degranulation, IGF transport and uptake by IGFBPs and others.

### TECs and NECs maintain their distinct gene expression patterns in ex vivo cell culture

**Figure 5:**
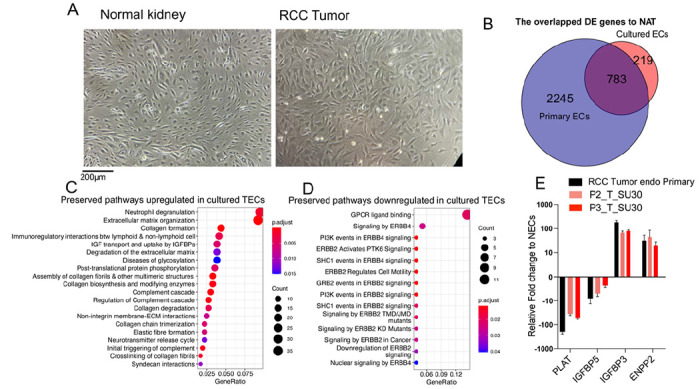
Phenotype and gene expression of *in vitro* cultured TECs and NECs **(A)** Cell morphology of cultured TECs and NECs at passage 3 in a 10X brightfield microscope. **(B)** Venn diagram of DEGs (padj < 0.05) in TECs compared with NECs and the overlap between cultured and primary ECs **(C, D)** Preserved pathways in cultured TECs derived from overlapping differentially expressed genes that are upregulated (log2FC ≥ 1) or downregulated (log2FC ≤ −1). **(E)** Key marker gene expression compared to NECs in the primary, passage 2, and passage 3 cultures.

A prerequisite to understanding the functional properties of unique cell types is to establish an *in vitro* culture system. Few previous studies of TECs have examined their properties *in vitro*, due to their slow growth and difficulty in maintaining viability. We were able to isolate primary NECs and TECs from 5 donors for successful *in vitro* culture for up to 2–3 passages in human VEGF-containing media (34, 44). TECs were larger, stellate, and adopted a lightly overlapping cobblestoned growth pattern *in vitro* while NECs were smaller, spindle shaped, and adopted a whorled arrangement in culture (Fig. 5A). We then generated bulk RNA-seq data from ECs at passage 3 as well as primary TECs and NECs freshly isolated (not cultured) from the same donors. We detected 1,002 DEGs between cultured TECs and NECs compared to 3,028 DEGs between primary isolated TECs and NECs. Comparison of the overlap of the DEGs between TECs and NECs showed that, although culturing ECs *in vitro* reduced the overall number of DEGs, 783 (78.1%) of cultured EC DEGs were still shared with the primary purified ECs (Fig. 5B).

To explore the core programs retained by TECs and NECs in culture, we performed pathway enrichment analysis of the shared DEGs. The major upregulated pathways that were conserved *in vitro* related to neutrophil degranulation, ECM organization, and regulation of interactions with immune/lymphoid cells (Fig. 5C). The major downregulated pathways preserved in TECs *in vitro* related to GPCR ligand binding, ERBB4 and ERBB2 signaling (Fig. 5D). Next, using quantitative RT-PCR, we validated our RNA-seq findings using key TEC and NEC marker genes previously identified (Fig. 5E). These experiments demonstrated that primary cultures of TECs and NECs could be established *in vitro* and retain key expression programs of their freshly isolated counterparts.

### TECs are resistant to VEGF withdrawal and exhibit distinct binding preferences for CD45^+^ leukocytes

**Figure 6:**
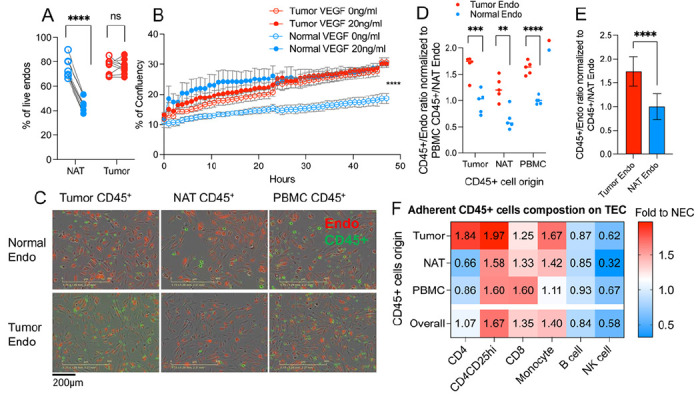
Co-culture of immune cells and *in vitro* established Ecs. **(A)** The percent of live EC (DAPI- population) after 48 hours of VEGF withdrawal (B) The percent of culture confluency over time. **(C)** Adherent leukocytes (green) and ECs (red) after 24 hours of co-culture. **(D)** Adherent CD45^+^ leukocyte / endothelial cell ratio normalized to PBMC CD45^+^ / NECs determined by flow cytometry after 24 hours of each co-culture combination. **(E)** Averaged adherent CD45^+^ leukocytes from different origins / EC ratio normalized to averaged CD45^+^ / NECs. **(F)** Adherent leukocyte composition on TECs. The percent of total leukocytes of each leukocyte population on TECs were compared to the corresponding % of population on NECs. Fold changes are displayed. Statistics: t-test, ** P ≤ 0.01, *** P ≤ 0.001, **** P ≤ 0.0001

Having established conditions for primary culture of TECs and NECs, we next explored their phenotypic and functional differences *in vitro*. Previous studies demonstrated that the survival of NECs from kidney in culture requires supplementation with VEGF in the culture medium (45). We first assessed whether TECs and NECs exhibited differential sensitivity to this important trophic factor by removing it from the culture. Within 48 hours of VEGF removal, NECs from 4 donors showed a sharp reduction in viability as assessed by DAPI flow staining (Fig. 6A). In contrast, TECs from those same 4 donors showed no loss in viability under the same conditions of VEGF withdrawal. We also performed live cell imaging of TECs and NECs over 48 hours (Fig. 6B). TECs showed a comparable increase in % confluency over the 48-hour assay period with or without the presence of VEGF in the culture medium. In contrast, the % confluency of NECs increased much more slowly without VEGF compared to the presence of VEGF. This differential sensitivity of TECs and NECs to VEGF may be related to the higher expression of KDR and other receptors for autocrine or serum-derived trophic factors both in the isolated and *ex vivo* cultured ECs (Suppl. Figure 4).

TECs represent the first barrier to entry of immune cells into the tumor mass and are an integral component of the tumor microenvironment. Immune regulatory pathways were altered in our analysis of DEGs both in freshly isolated (Fig. 1) and cultured TECs (Fig. 5). This observation led us to empirically test whether CD45^+^ leukocytes exhibited different adherence to TECs versus NECs. We isolated CD45^+^ leukocytes from the tumor mass, NAT or peripheral blood mononuclear cells (PBMCs). We applied fluorescent labeled leukocytes to autologous labeled TECs or NECs in the IncuCyte imaging platform (Figure. 6C). After 24 hours of co-culture, we enumerated bound CD45^+^ leukocytes and normalized that count versus the number of TECs or NECs in the culture (Fig. 6D). Regardless of their origin (tumor, NAT, PBMC), more CD45^+^ leukocytes adhered to TECs compared to NECs (Fig. 6E). We then used flow cytometry to determine if specific CD45^+^ leukocyte subsets were responsible for this difference (Suppl. Figure 6A). We determined the relative fold binding of specific leukocyte subsets as a function of their origin (Fig. 6F, Suppl. Figure 6B). Regardless of their tissue of origin, T-cells and monocytes but not B cells and NK cells were more likely to interact with TECs. Although they were few in number, CD4^+^CD25^+^ T regulatory cells were much more likely to adhere to TECs versus NECs. These results may provide an explanation for the preferential recruitment of T-cells and monocytes into the RCC tumor microenvironment that has been reported previously (46). Taken together these studies demonstrated the utility of *in vitro* culture of TECs and NECs to study their distinct functional and immunological properties.

## Discussion

TECs are an important component of the tumor microenvironment and represent a contact interface between the circulation and the tumor. Since ECs comprise a small proportion of cells in a tissue, studies without enrichment will be underpowered to detect their heterogeneity. In comparison to the previous ccRCC scRNA-seq studies (12, 42), we provide the deepest characterization of enriched TECs, and matched NECs reported to date. This allowed identification of selective *IGFBP3* expression in RCC TECs, as well altered expression of ECM regulation pathways, which were not detected in prior studies. IGFBP family members show selective expression in various NEC types from kidney (47). Of these, *IGFBP3* is very focally expressed in peritubular capillary ECs in normal kidney (48), whereas it is broadly expressed in TECs in HCC (15) and ccRCC (15). One possible mechanism for *IGFBP3* induction in TECs may be the hypoxic tumor microenvironment, since it is a HIF-regulated gene (49–51). *IGFBP3* maintained increased expression in TECs compared to NECs, even when both were cultured under *ex vivo* hypoxic conditions (1% O_2_). IGFBP3 can promote sprouting angiogenesis (52–54), and retinal neovascularization (55), and the proportion of TECs with an angiogenic phenotype is thought to correlate with susceptibility to anti-VEGF therapy (43). This correlates with our observations of the relatively high proportion of TECs with an angiogenic phenotype in RCC (Fig. 1), and the negative prognosis of high *IGFBP3* expression in RCC (Suppl. Figure. 2). However, direct targeting of IGFBP3 as a common TEC-directed therapy may not be straightforward since it appears to have tumor-promoting and anti-tumor effects in different cancers (56). While it is intriguing that a recent study showed that direct blockade of IGFBP3 may provide a new therapeutic avenue in RCC (57), this is tempered by lack of efficacy of blockade of IGFBP3’s receptor IGF1R in recent clinical trials (58). In addition, IGFBP3 may have roles beyond its binding to IGF1R, and therefore further study of its function in RCC TECs is warranted.

Another advance in our study is the description of molecular phenotypes that are retained by TECs and NECs in *ex vivo* culture. We found that TECs were more resistant to cell death after withdrawal of VEGF. This finding is consistent with a previous study that reported proangiogenic properties and resistance of RCC TECs to vincristine-induced apoptosis compared to NECs (59). Analysis of retained expression signatures showed that TECs had altered expression of pathways regulating the ECM, IGF pathways and immunoregulation in *ex vivo* culture. We leveraged our ability to study these properties since TECs have been proposed to have multiple immunomodulatory roles in tumors such as anergy induction, antigen presentation and secretion of factors that affect T-cell migration and priming (60). Our experiments exploring the interaction of autologous immune cell subsets with ECs demonstrated that CD8^+^ T-cells and monocytes increasingly adhere to TECs compared with NECs, which may contribute to the observation that ccRCC tumors often show increased infiltration of T cells and monocytes compared to normal kidney tissue (46, 61). We also found that CD4^+^CD25^+^ cells are able to preferentially adhere to TECs regardless of their tissue of origin (tumor, normal kidney tissue or peripheral blood). The presence of increased numbers of immunosuppressive CD4^+^CD25^+^ regulatory T-cells and exhausted CD8^+^ T-cells within RCC tumors explains the efficacy of immune checkpoint inhibitors in overcoming immune tolerance of the tumor (62, 63). Our results show that the increased frequency of immunosuppressive regulatory T-cells in RCC tumors is likely to be established at the point of T-cell entry into the tumor mass via interactions with TECs. Future studies will explore the interactions of leukocytes with TECs and NECs to assess their penetration into physiologically relevant 3D tumor culture environments (64).

In summary, we investigated a deep reference dataset of isolated and purified TECs from RCC and matched NECs from adjacent kidney tissue. Compared to NECs, TECs had altered expression of genes related to regulation of ECM, IGF signaling and cell-cell interactions, many of which were stably retained in *ex vivo* primary culture. By comparing EC expression signatures across kidney and liver cancers, we found that while organ-specific NECs were heterogeneous, the majority of TECs shared a common phenotype. We also demonstrate increased interaction of immune suppressive T regulatory cells with TECs providing a mechanism for their enhanced entry into RCC tumors. These findings advance our understanding of TECs in RCC and provide opportunities to exploit or modify their phenotypes as a therapeutic strategy.

## Data Availability

Sequencing data have been deposited in the National Center for Biotechnology Information Gene Expression Omnibus (scRNAseq: GSE237425, RNAseq: GSE237427). Original script is available athttps://github.com/YuexinXu/RCC_Endo. The interactive scRNAseq dataset of RCC and HCC endothelial populations created by Shiny and ShinyCell (65) R packages are available at https://yxu2.shinyapps.io/shinyapp/.
